# Model variations in predicting incidence of *Plasmodium falciparum *malaria using 1998-2007 morbidity and meteorological data from south Ethiopia

**DOI:** 10.1186/1475-2875-9-166

**Published:** 2010-06-16

**Authors:** Eskindir Loha, Bernt Lindtjørn

**Affiliations:** 1 Department of Public and Environmental Health, Hawassa University, Ethiopia; 2 Centre for International Health, University of Bergen, Norway

## Abstract

**Background:**

Malaria transmission is complex and is believed to be associated with local climate changes. However, simple attempts to extrapolate malaria incidence rates from averaged regional meteorological conditions have proven unsuccessful. Therefore, the objective of this study was to determine if variations in specific meteorological factors are able to consistently predict *P. falciparum *malaria incidence at different locations in south Ethiopia.

**Methods:**

Retrospective data from 42 locations were collected including *P. falciparum *malaria incidence for the period of 1998-2007 and meteorological variables such as monthly rainfall (all locations), temperature (17 locations), and relative humidity (three locations). Thirty-five data sets qualified for the analysis. Ljung-Box Q statistics was used for model diagnosis, and R squared or stationary R squared was taken as goodness of fit measure. Time series modelling was carried out using Transfer Function (TF) models and univariate auto-regressive integrated moving average (ARIMA) when there was no significant predictor meteorological variable.

**Results:**

Of 35 models, five were discarded because of the significant value of Ljung-Box Q statistics. Past *P. falciparum *malaria incidence alone (17 locations) or when coupled with meteorological variables (four locations) was able to predict *P. falciparum *malaria incidence within statistical significance. All seasonal AIRMA orders were from locations at altitudes above 1742 m. Monthly rainfall, minimum and maximum temperature was able to predict incidence at four, five and two locations, respectively. In contrast, relative humidity was not able to predict *P. falciparum *malaria incidence. The R squared values for the models ranged from 16% to 97%, with the exception of one model which had a negative value. Models with seasonal ARIMA orders were found to perform better. However, the models for predicting *P. falciparum *malaria incidence varied from location to location, and among lagged effects, data transformation forms, ARIMA and TF orders.

**Conclusions:**

This study describes *P. falciparum *malaria incidence models linked with meteorological data. Variability in the models was principally attributed to regional differences, and a single model was not found that fits all locations. Past *P. falciparum *malaria incidence appeared to be a superior predictor than meteorology. Future efforts in malaria modelling may benefit from inclusion of non-meteorological factors.

## Background

Over 100 million people worldwide are affected by malaria and *P. falciparum *malaria is responsible for approximately one million deaths annually, with many of those deaths occurring in children under the age of five years. Unfortunately, 90% of the global malarial burden is carried by sub-Saharan Africa [1,2]. Malaria transmission is complex and not yet fully understood; the recent focus of developed nations on global warming has spawned the suspicion of a climate-malaria link.

The possible association of changes in temperatures to variations in malaria epidemiology is merited by the well-defined biological effects on life-cycle stages of the *Anopheles *insect transmission vector and the *Plasmodium *malaria parasite [3]. For example, increasing of the temperature to 31°C results in a shortened sporogonic period of the *Plasmodium *parasite, an effect which differs among the *P. falciparum *and *P. vivax *species. Higher mean daily temperatures are not favourable for vector survival since increased temperatures speed up development of the aquatic stages of the vector's life cycle.

Many researchers, therefore, have proposed developing improved tools to forecast malaria epidemics by using variations in regional temperatures. These efforts have resulted in the medical literature using vastly inconsistent terminology to describe malaria risks, and to distinguish between long-term forecasts, early warning and early detection of epidemics.

Long-term epidemic forecasting is usually based on climate forecasting, and relies on such datasets as the El Niño Southern Oscillation indices to predict epidemic risk months in advance over large geographical areas. Such a forecast allows time for the population to prepare for a possible epidemic in the upcoming malaria season.

Malaria epidemic early warning is based on surveying transmission risks to predict timing of an increase based on abnormal rainfall or temperatures. Often, such risks are influenced by population vulnerability, such as history of low rates of malaria transmission. Such predictions of malaria epidemics can provide lead times of weeks to months.

The long-term and early warning approaches should, however, be distinguished from epidemic early detection, which involves noting the beginning of an unusual epidemic. As such, this surveillance approach is limited in that is offers little lead time (days to weeks) for preparation and implementation of preventive measures. When used in an effective manner, it is able to prevent sickness and death.

The aim of this study was to examine if the spatio-temporal distribution of surface temperature and rainfall are useful factors to predict changes in malaria incidence, as a malaria epidemic early warning strategy. This evaluation was based on an assumption that the link between climate and occurrence of malaria is constant and similar for different regional settings.

Incorporating prediction and forecasting approaches, however, calls for sound understanding of the complex factors involved in malaria transmission. It has been suggested that the major driving force of malaria transmission is climate [4-8]. However, the data has been largely inconsistent as to exactly how climate influences malaria transmission. In some geographic regions, the minimum temperature has been shown as an important contributory factor for malaria transmission [6,7,9], while in others the maximum temperature has been implicated [5,10-12]. Onset of malaria epidemics often coincide with the rainy season or significant rainfall [13,14], but this is not always the case [9]. Inconsistent findings also exist in studies focusing on the number of climate lags, both for rainfall and temperature, associated with malaria epidemics [5-7]. Prediction strategies based on climate information have been most accurate when considering colder locations [6]; still, not all studies have been able to confirm the utility of meteorological variables at varying altitudes [7]. The effect of rainfall on transmission rates has also been found to vary between urban and rural areas [6], suggesting the presence of an additional cofounding factor in one of both of these communities. To date, many different models have been developed based on the simple assumption that a defined set of climatic variables influence malaria incidence; however, the models have different statistical or mathematical forms, incorporate different variables and lag combinations, and demanded different forms of data transformation and analysis [5-8]. This might reflect the complexity between climatic variables and malaria transmission [15] while striving to address biological plausibility. Unfortunately, such biological approaches are able to describe malaria transmission but are not powerful enough to yield reliable predictions of incidence [16]. This is also a limitation that affects implementation of climate-based malaria early warning and forecasting [17]. Attempts have been made to improve the models using historical morbidity and climatic variables [6-8,12].

Nonetheless, the impact of climate on malaria transmission has yet to be firmly established. Thus, there exists a need to consider local variations in climates in order to fully understand the relationship between climate and malaria transmission [11,15,16,18-23]. Taking the average of conditions across large geographic areas, or even making similar assumptions stating the effects of climatic variables to be constant across different locations [7], might cause an underestimation of local variations and disable a models accurate ability to predict malaria incidence [16,17].

In addition to the incorporation of climatic causes, some researchers have suggested building models that consider non-climatic factors such as land use, population movement, immunity, topography, parasite genotypes, vector composition, drug resistance, vector control measures and availability of healthcare services [6-8,18,20,24]. The study presented herein did not include non-meteorological data because of limited data availability; however, local variations were considered in the link between malaria incidence and meteorological factors [10,18,20]. In areas such as the Ethiopian highlands endemic malaria occurs at altitudes above 2100 m [25]. It has been suggested that global warming will drive malaria transmission at higher altitudes, mainly because of corresponding changes in the distribution of the *Anopheles *vector [26]. However, the study period covered less than 10 years and, thus, was too brief to evaluate the potential effects of global warming on vector distribution and on malaria incidence. Therefore, the principal objective of this study was to explore if variations of meteorological factors are able to consistently predict *P. falciparum *malaria incidence at different locations in south Ethiopia.

## Methods

### Data inputs and inclusion criteria

A total of 42 locations in the southern region of Ethiopia were examined for data on varying serial length of *P. falciparum *malaria incidence during 1998-2007; available data from local meteorology stations were also collected for the analysis. The minimum serial length was set at 50 [27], resulting in five locations being excluded from further analysis. To ensure against imputation effects on model structure, the threshold of allowable missing data was set at 15% of the total series; this criterion led to the exclusion of two locations. The final number of locations in this study was, therefore, 35.

**Figure 1:**
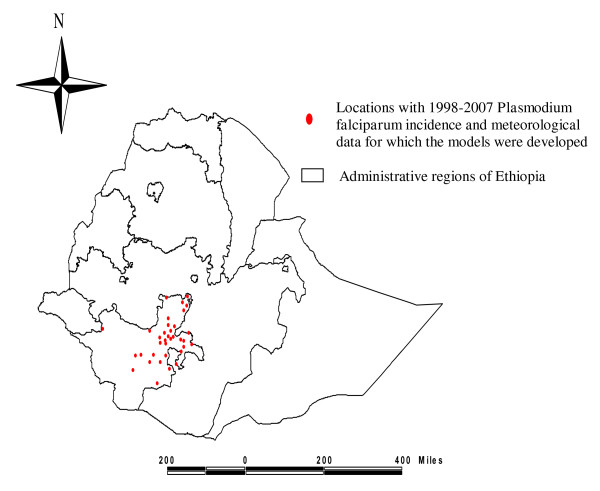
**Coordinates of the malaria affected locations of interest in south Ethiopia**. A map of Ethiopia has been sub-divided into administrative regions that include the Southern Nations and Nationalities People's Region where we conducted this study.

Microscopically-confirmed *P. falciparum *malaria cases only were considered in this study. The total number of cases was 210 659 and covered a period of 6.7 years from the 35 locations. The mean serial length was 80 months (range: 51-118 months). The available meteorological data included: monthly total rainfall in millimetres (all locations), monthly average maximum, minimum and average temperature in Celsius (17 locations) and monthly average relative humidity as percentage (three locations). While taking averaged rainfall data for each month in the series, 19 locations exhibited a unimodal rainfall pattern (which peaked from June to September) and the remaining 16 locations exhibited a bimodal pattern (peaks in February to April and August to October). The bimodal rainfall pattern could 'double' the serial length and, hence, improve the chance of observing any link between *P. falciparum *malaria incidence and rainfall pattern. The altitude of the meteorology stations ranged from 1182-2582 m, and 14 locations were below 1750 m. Figure 1 presents the coordinates of each location.

### Data source

A health centre provides basic curative and preventive health services for a population of about 25,000 people. Each health centre is staffed by nurses and health officers, and by trained laboratory technicians. The institutions routinely performed thick and thin blood film examinations for malaria parasites. Rapid diagnostic tests for malaria were not used. Each month, all health institutions reported suspected malaria cases and confirmed *P. falciparum *and *P. vivax *cases to the regional health authorities.

Microscopically-confirmed *P. falciparum *malaria cases (*n *= 210,659) were obtained from the reports made to the Southern Nations and Nationalities Regional Health Bureau from 33 health centres and from two district hospitals.

The meteorological data used for analyses were obtained from the Southern Branch office of the National Meteorological Agency of Ethiopia. This agency operates over 200 meteorological stations, with records spanning 15 to over 50 years. From the year 1970 onward, the proportion of missing data is low [28]. In this study, meteorological data from 35 locations was used (Figure 1).

### Missing data handling

The Box-Jenkins method [29] was used, hereafter referred to as the Autoregressive integrated moving average (ARIMA). As this model requires discrete time series data with no missing values, missing data replacement for both dependent and independent series was carried out. Missing values were replaced with the mean or the median of the period in which the observation was missing. The mean was used for data of *P. falciparum *malaria incidence, temperature and relative humidity. Since rainfall data was heavily skewed, the median was used to replace the missing values of rainfall.

### Assumptions

1. The underlying data of malaria transmission was assumed to be stochastic, whereby local variations and other unmeasured causes play important roles. Others have reported local variations in the association between climate and malaria incidence [15,16,18-22]. Therefore, building models for each location would remove crudeness of models and enable reliable forecasts. In addition, this might contribute to our understanding of the impact of meteorology with respect to varying altitudes.

2. The quality of data obtained through routine reporting in developing countries may be questionable, mainly because of under-reporting. However, the data sets were assumed to hold the basic elements of malaria transmission like trend, seasonality or monthly variations, which could suffice for modelling exercise [12]. The need to use available data at the regional level, check whether these data could be used to model *P. falciparum *malaria incidence and assist malaria epidemic early warning was considered.

3. The meteorology station correctly captures climate data within a 10 km radius [Southern Branch office of National Meteorological Agency of Ethiopia, personal communication], and this matches to the service area coverage of the corresponding health centre. This assumption does not apply for the two district hospitals since the service area coverage of a district hospital is beyond the 10 km radius [30].

4. In Ethiopia, malaria transmission is largely unstable [31] and, hence, the population has insignificant immunity, putting all age groups at equal risk of contracting the disease. Therefore, demographic changes were assumed to have had minimal impact in malaria transmission during the study period. Meanwhile, there is lack of proper denominator for health facility-based data in this country. As a result, the number of malaria cases was used instead of its fraction out of the total population.

### Scope

This paper sought to unveil the local variations in the predictive power of lagged effects of the number of past *P. falciparum *malaria cases and climatic variables on incidence of *P. falciparum *malaria. As model structures of each location were presented, detail of each model, forecasting and validation were beyond the scope of this research effort.

### Data processing and analysis

SPSS version 17.0 Expert Modeler (Chicago, IL, USA) was used to automatically determine the best-fitting model. Malaria incidence was the dependent variable, and all available climatic variables were fed into the model as predictors. The Expert Modeler keeps the predictor series in the model only if it is significant. The resultant model was checked for consistency by inserting the model criteria set and significant predictor identified by the Expert Modeler. To do this, custom ARIMA models were used and several logical combinations of criteria to look for better models were considered. The best-fitting model built by the Expert Modeler was subsequently used. For the locations of Cheleklektu and Buee, a constant value of 1 was added to the dependent series to enable log transformation. Automatic detection of outliers was made and the outliers were modelled accordingly, thus trimming was not performed. The same procedure was followed for all data sets.

### Goodness of fit

The R-squared measurement was used as an indicator of goodness of fit for the models if there was no differencing. The R-squared coefficient of determination suggests the proportion of variance of the dependent variable explained by the model. The stationary R-squared was used instead whenever the Expert Modeler considered differencing. The stationary R-squared was used to capture trend or seasonality, which is the basis for differencing. The stationary R-squared and the ordinary R-squared values were the same when there was no data transformation to any form. It is noted that if the series was log transformed without differencing, stationary R-squared would overestimate the ordinary R-squared and underestimate for the square root transformation.

### Diagnostic statistics

The Ljung-Box Q statistic, also known as the modified Box-Pierce statistic, was used to provide an indication of whether the model was correctly specified. A significant value less than 0.05 was considered to acknowledge the presence of structure in the observed series which was not accounted for by the model; therefore, we ignored the model if it had significant value.

The residual autocorrelation function was expected to agree with the white noise assumption. White noise, the most common model of noise in time series analysis, is a stationary time series or a stationary random process with zero autocorrelation. In other words, in white noise *N*(*t*) any pair of values *N*(*t*_*1*_) and *N*(*t*_*2*_) taken at different moments *t*_*1 *_and *t*_*2 *_of time are not correlated; that is, the correlation coefficient *r*(*N*(*t*_*1*_), *N*(*t*_*2*_)) is equal to null. The SPSS 17.0 forecasting menu provides autocorrelations that provides *p *values for each lagged noise residual series using the Ljung-Box statistics. It was possible to see which lagged noise residual was significantly autocorrelated. For each data set, autocorrelation of noise residuals was carried out, and the results were consistent with that of the model statistics table of Ljung-Box Q statistics.

### The model

Since meteorological variables were used as predictors, addition of the Transfer Function (TF) model to the basic univariate ARIMA model was considered. Whenever the Expert Modeler dropped the predictor series, the model was found to take on the univariate ARIMA form.

### ARIMA orders

In ARIMA (*p, d, q*) (*P, D, Q*), the first parenthesis held the non-seasonal autoregressive (*p*), differencing (*d*) and moving average (*q*) orders. A non-seasonal autoregressive order of 1 specified the value of the series in one time period from the past to be used to predict the current value. A first order differencing implied a linear trend in the series. Meanwhile, moving average order of 2 specified the deviations from the mean value of the series from each of the last two time periods to be considered when predicting the current value. The second parenthesis held their seasonal counterparts. For monthly data, the seasonal order of 1 implied that the current value was affected by the series value 12 periods prior to the current one.

### Transfer function orders

*Numerator *specified which previous values from the predictor series were to be used to predict the current value of the dependent series.

*Denominator *specified how deviations from the series mean, for previous values of the predictor series, were to be used to predict the current value of the dependent series.

*Difference *specified the order of differencing applied to the predictor series before estimating the model occurred.

The seasonal orders were built using the same strategy as that for the ARIMA orders.

### Delay

Setting a delay is known to cause the predictor's influence to be delayed by the number of intervals specified. For instance, a delay of 4 implies the value of the predictor at time *t *does not affect forecasts until four periods have elapsed (*t *+ 4). A delay of 4 essentially equals lag of four time periods.

See Additional file 1 for details of the model.

### Data transformation

The ARIMA model is an analysis in the temporal domain applied to stationary data series. Thus, the presence of outliers, random walk, drift, trend, or changing variance in the series might have resulted in nonstationarity. And the stationarity of the series could be achieved when both the mean and the variance remained constant over time. For this, variance stabilizing transformations, like natural log (LN) and square root (SQR), and detrending using differencing were used when necessary. In addition, the Expert Modeler was set to detect outliers (if any) and model them automatically.

## Results

### Model inclusion and exclusion

Data from 35 locations were analysed using Time Series modelling. Models of five locations were ignored because of the significant results of the diagnostic statistics, the Ljung-Box Q, including models built for the two hospital locations.

### Data description

We analysed 210 659 microscopically-confirmed *P. falciparum *malaria cases from 35 localities (Figure 1). During the same period, these institutions also reported 112 354 microscopically-confirmed *P. vivax *malaria cases. The ratio of *P. falciparum *to *P. vivax *malaria cases was 1.87 to 1.00.

The pattern of meteorological variables and *P. falciparum *malaria monthly cases was not uniform across the locations, indicating local variations. Sequence charts were generated for each of the 35 locations and for the mean meteorological conditions of 23 (rainfall) and 14 locations (rainfall and temperature). The lagged effect of rainfall on *P. falciparum *malaria incidence was more visible for the mean meteorological conditions (Additional file 2).

### Past *Plasmodium falciparum *malaria incidence

Of 30 models, 21 were based on lagged effect of incidence data alone (17 locations) or coupled with meteorological predictors (4 locations). Among those 21 models, 16 had a non-seasonal AR order of 1 (13 locations) or 2 (3 locations). Three locations had both seasonal and non-seasonal AR orders of 1. Two locations had only a seasonal AR order of 1. Non-seasonal and seasonal first order differencing was used for five and three locations, respectively. Five locations had a non-seasonal MA order of range 1-6, and there was no seasonal MA order. Seasonal ARIMA orders were specified for six locations of altitude 1742 m or higher, constituting one-third of the locations above this altitude (Additional file 3, Tables S1-S4).

### Meteorological data

Rainfall data were available from all locations, however, it was found to be a significant predictor for only four of the locations (altitude: 1182, 1431, 1618 and 2054 m). A delay of 2 months was significant for 2 of these. A delay of 2 months with numerator TF order of 0 refers to a 2 months lagged effect (Additional file 3, Table S1). Besides the delay value of 2, including the numerator TF orders of 2, 1 and 0 was interpreted as the rainfall data corresponding to the previous four, three and two months to predict the current incidence. The denominator TF orders of 2 and 1 indicated that the model used the deviations of rainfall data of the 4^th ^and 3^rd ^lagged months (delay 2) from the series mean. One model specified numerator TF order of 2, 1 and 0 without setting a delay; that is, rainfall data of the last two consecutive months coupled with the current one were used to predict incidence (Additional file 3, Table S2). A single location had 3 months lagged effect of rainfall (Additional file 3, Table S4).

Minimum and maximum temperatures were available for 17 of the locations. Minimum temperature was found to be a significant predictor in five locations. Delays of 2, 4 and 5 months with numerator TF order 0 predicted incidence in three locations (altitudes: 2582, 1220 and 2331 m). Of those, first order non-seasonal differencing was required for the location with the lowest altitude (1220 m). Incidence (two locations) and maximum temperature (one location) were included in the models (Additional file 3, Tables S1 and S4). For the remaining two locations, numerator TF order of 0 without a delay was specified; that is, the current value of the predictor was used to determine the current incidence with no contribution for forecasting. For one of the models, however, it impacted incidence to achieve higher goodness of fit statistics. The other model had the worst goodness of fit statistic (negative value) of all models (Additional file 3, Table S2).

Maximum temperature at a lag of 4 months coupled with the deviations of a lag of 5 and 6 months from the series mean predicted incidence at an altitude of 1221 m (Additional file 3, Table S1). Meanwhile, coupled with minimum temperature, the value at lag of 4 when added to the lag of 2 months predicted incidence (Additional file 3, Table S4).

Only three locations had data available on relative humidity, but none proved significant.

### Goodness of fit of models

Except for one model which produced a negative value, the range of the R-squared was 16-97%. Of 30 models, 20 had values greater than 50% and seven had values exceeding 85%. The range for models with any of the seasonal ARIMA orders was 60-97%. The models were reasonably good for explaining the total variations of the data sets. According to the Spearman's rho correlation coefficient, there was no significant correlation between the R-squared values and the serial length (r = 0.29) or the average incidence per month (r = -0.01).

### Model similarities and variations

The model predicted incidence fairly well by its lagged values in most locations. Models of seven locations were similar with ARIMA (1, 0, 0) (0, 0, 0) with no transformation. Nevertheless, the other incidence models applied different forms of transformation (LN, SQR or differencing) or incorporated different meteorological variables. Some models did not contain incidence at any AR or MA orders. Meanwhile, meteorological variables were significant predictors for only seven of the locations without any apparent reiteration in line with the altitude. For two of the data sets, the Expert Modeler revealed the absence of a significant predictor with reasonable goodness of fit statistics. And for five of the data sets, the model did not comply with the criterion of diagnostic statistics. In summary, the variations outweighed the similarities of the models made for different locations for the given incidence and meteorological data.

### Mean meteorological conditions

It was not possible to engage all (thirty) data sets to evaluate the utility of taking mean meteorological conditions for prediction because aggregates of *P. falciparum *malaria incidence of all data sets produced a serial length of 29 (below 50). Therefore, the mean condition of 23 locations with a serial length of 62 was used. This resulted in monthly rainfall being a significant predictor at a lag of 4 months coupled with AR order of 1 (monthly rainfall was a significant predictor only in four locations when the data sets were analysed separately). This model was applied to each of the 23 locations but did not produce any significant results. The mean condition of temperature (for 14 locations) with or without rainfall was also checked, but the diagnostic statistics disqualified the model (Additional file 3, Table S4).

## Discussion

Statistical modelling is used for understanding and prediction of multifactorial based events; as such, reproducibility, biological plausibility and robustness govern the applicability and effectiveness of each resultant model. Malaria transmission is one such complex event as many underlying causes have been associated with its frequency and duration, including regional factors. The Malaria Early Warning System (MEWS) has been established to enable reliable predictions of *P. falciparum *malaria epidemics using transmission risk indicators like unusual increase in rainfall [32]. Nevertheless, producing accurate predictions using climate data remains a challenge [15]. This study set forth to evaluate large populations affected by malaria and residing in widely variable geographical areas; specifically, we considered local variations to determine their contribution and ability to limit effective prediction of *P. falciparum *malaria incidence by using statistical modelling methods. This strategy is expected to also provide evidence to support the meteorology-malaria link and to determine the validity of using mean meteorological conditions to create a general predictive model.

The Ljung-Box Q provided the diagnostic statistics to check the presence of structure in the observed series which was not accounted for by the model. Five models were ignored with significant values according to this diagnostic statistic, but the underlying reasons were not immediately clear. It was likely that the only two data sets that came from hospitals might not have properly coincided with the station-specific climate data since the catchment area of those hospitals was wider than that of the meteorological stations. Thus, it remains to be seen whether linking hospital data with wider catchment to station-specific meteorological data would benefit evaluations of the proposed meteorology-malaria link.

Malaria transmission is known to be associated with gametocyte prevalence in a population [33]. The finding that past *P. falciparum *malaria incidence often predicts the current incidence may support this relationship. Likewise, the AR term in this study was found to be much more important than the meteorological variables for prediction of *P. falciparum *malaria. Other studies have also shown that models using the previous month's incidence for malaria prediction outweigh the impact of climate variables [6-8,12]. In this study, the MA order was also used. Seasonal ARIMA orders were specified at higher altitudes only, starting at 1742 m. This might reflect the seasonal nature of malaria transmission in these locations. However, two-thirds of the locations above this altitude did not have seasonal ARIMA orders. So, it was not possible to conclude that malaria was seasonal at areas above the altitude of 1742 m, or that erratic rainfall patterns or other unmeasured potentially confounding factors have eluded the seasonality.

As has been shown by others [34,35], this study confirms the biologically driven link of temperature and rainfall with malaria transmission. However, the link is complex and sensitive to the effects of other factors, and it remains to be seen whether direct and predictable relationships really exist [36]. This study suggests that temperature and rainfall are significant predictors for only a few of the locations examined. The absence of temperature data for 13 of the locations might have limited our findings. A serial length above the minimum requirement for the ARIMA models was used, but it might not have had enough statistical power to yield significant values or be long enough in duration to capture seasonality. Nevertheless, the rainfall pattern was bimodal in 16 out of 35 locations, offering a better chance to see the effect of rainfall on *P. falciparum *incidence. Some researchers have suggested the use of weekly data to model malaria incidence [5,7,12]. Thus, the monthly morbidity and meteorology data used in this study might not be sensitive enough to reveal the association between meteorology and *P. falciparum *malaria incidence.

Considering mean conditions or aggregated data might disguise real effects [16,17]. This urges approaches of modelling to specific locations at the expense 'all-fit-one' or simple model. This study also examined the effect of mean meteorological conditions and compared it with the area-specific results. Meteorological variables, particularly rainfall, were able to predict *P. falciparum *malaria incidence on a wider geographic scale. However, the local variations seen in the link between *P. falciparum *malaria incidence and meteorological factors have not allowed such approaches of prediction. More importantly, if malaria transmission cannot be explained by the meteorology-malaria link, it is necessary to include non-climatic factors like vector composition, vector control measures and healthcare services in statistical modelling, as is advocated by others [6-8,18,20,24,37].

## Conclusions

This study shows that models of climate-malaria link varied from place to place, and one model could not fit all locations. In several locations, it was found that past *P. falciparum *malaria incidence was a more robust predictor than any of the meteorological variables. It is possible that more accurate malaria modelling may require the inclusion of non-climatic causes as well. Nonetheless, statistical time-series modelling to analyse meteorology-malaria link appears to be a promising approach to predicting malaria incidence and merits further investigation.

## Competing interests

The authors declare that they have no competing interests.

## Authors' contributions

EL conceived the study, collated, analysed and interpreted the data, and prepared the draft manuscript. BL conceived the study, guided the analysis, interpreted the data and helped to draft the manuscript. Both authors have read and approved the submitted version of the manuscript.

## Supplementary Material

Additional file 1 **Details of the model**. Formulae and main features of Transfer Function and univariate ARIMA models. Click here for file

Additional file 2 **Sequence charts**. Sequence charts for each of the 35 locations examined, mean meteorological conditions of 23 and 14 locations. The separate sheets in the Excel file are labeled by the name of the locations corresponding to the data. Data displayed include altitude, available meteorological variable(s) and *P. falciparum *incidence. Click here for file

Additional file 3 **Tables S1 to S4: Time series models to predict *Plasmodium falciparum *malaria incidence at different locations in south Ethiopia**. The 35 locations were divided among four tables for ease of presentation. All tables included data on location, altitude, available data used, model structure, goodness of fit, significant variables and model description, serial length and average incidence per month. Click here for file
