# Supplementary material for: Model variations in predicting incidence of Plasmodium falciparum malaria using 1998-2007 morbidity and meteorological data from south Ethiopia
Source: Malar J. 2010 Jun 16;9:166. doi: 10.1186/1475-2875-9-166 (PMC2898788; doi:10.1186/1475-2875-9-166)
Supplement: Additional file 1 — Details of the model. Formulae and main features of Transfer Function and univariate ARIMA models. [file 1475-2875-9-166-S1.PDF]

Additional file 1: Details of the model

Notations used :

$a_t\,(t=1,2,...,n)$ White noise series normally distributed with mean zero and variance σ^2

p Order of the non - seasonal autoregressive part of the model

q Order of the non - seasonal moving average part of the model

d Order of the non - seasonal differencing

P Order of the seasonal autoregressive part of the model

Q Order of the seasonal moving average part of the model

D Order of the seasonal differencing

s Seanality or period of the model

$\phi_p(B)$ AR polynomial of B of order $p, \phi_p(B) = 1 - \varphi_1 B - \varphi_2 B^2 - ... - \varphi_p B^p$

$\theta_q(B)$ MA polynomial of B of order $q, \theta_q(B) = 1 - \vartheta_1 B - \vartheta_2 B^2 - ... - \vartheta_q B^q$

$\Phi_P(B^s)$ Seasonal AR polynomial of BS of order $P, \Phi_P(B^s) = 1 - \Phi_1 B^s - \Phi_2 B^{s2} - ... - \Phi_P B^{sP}$

$\Theta_Q(B^s)$ Seasonal MA polynomial of BS of order $Q, \Theta_Q(B^s) = 1 - \Theta_1 B^s - \Theta_2 B^{s2} - ... - \Theta_Q B^{sQ}$

Δ Differencing operator $\Delta = (1-B)^d (1-B^s)^D$

B Backward shift operator with $BY_t = Y_{t-1}$ and $Ba_t = a_{t-1}$

$Z\sigma_t^2$ Prediction variance of Z_t

$N\sigma_t^2$ Prediction variance of noise forcasts

A Transfer Function model describing the relationship between the dependent and predictor series has the following form:

$$Z_t = f(Y_t)$$

$$\Delta Z_t = \mu + \sum_{i=1}^{k} \frac{Num_i}{Den_i} \Delta_i f_i(X_{it}) + \frac{MA}{AR} a_t \quad \textbf{(1)}$$

Droping the predictor series yields univariate ARIMA:

$$\Delta Z_t = \mu + \frac{MA}{AR} a_t \quad \textbf{(2)}$$

Main features of the model :

- Initial data transformations of the dependent and predictor series
- A constant term μ
- The unobserved i.i.d., zero mean, Gaussian error process a_t with variance σ^2
- The moving average lag polynomial $MA = \theta_q(B)\Theta_Q(B^s)$ and the auto - regressive lag polynomial $AR = \phi_p(B)\Phi_P(B^s)$
- The difference/lag operators Δ and Δ_t
- Predictors are assumed given. Their numerator and denominator lag polynomials are of the form :

  $Num_i = (\omega_{i0} - \omega_{i1} B - ... - \omega_{iu} B^u)(1 - \Omega_{i1} B^s - ... - \Omega_{iv} B^{vs}) B^b$ and

  $Den_i = (1 - \delta_{i1} B - ... - \delta_{ir} B^r)(1 - \Delta_{i1} B^s - ...)$
- The "noise" series

  $N_t = \Delta Z_t - \mu - \sum_{i=1}^{k} \frac{Num_i}{Den_i} \Delta_i X_{it}$ is assumed to be a mean zero, stationary ARMA process.
