# Supplementary material for: Model variations in predicting incidence of Plasmodium falciparum malaria using 1998-2007 morbidity and meteorological data from south Ethiopia
Source: Malar J. 2010 Jun 16;9:166. doi: 10.1186/1475-2875-9-166 (PMC2898788; doi:10.1186/1475-2875-9-166)
Supplement: Additional file 3 — Tables S1 to S4: Time series models to predict Plasmodium falciparum malaria incidence at different locations in south Ethiopia. The 35 locations were divided among four tables for ease of presentation. All tables included data on location, altitude, available data used, model structure, goodness of fit, significant variables and model description, serial length and average incidence per month. [file 1475-2875-9-166-S3.PDF]

Table S1: Time series models to predict *Plasmodium falciparum* malaria incidence at different locations in south Ethiopia

Location (Health centre, unless specified)	Altitude	Available data used (Temperature- Max and Min)	Model Structure (ARIMA)	Goodness of fit (R^2, if *: stationary R^2)	Significant Variables and model description	Serial Length	Average Incidence per month
Daramalo Wacha	1182	Incidence<br>Rainfall	(1,0,0)(0,0,0)	0.56	**Incidence** (SQR) at lag 1<br>**Rainfall** at delay 2 and numerator TF order of 0.	68<br>(Mar 2002-Oct 2007)	45
Tepi	1205	Incidence<br>Temperature<br>Rainfall	(1,0,0)(0,0,0)	0.30	**Incidence** (LN) at AR lag 1	117<br>(Jan 1998-Sep 2007)	57
Arba Minch	1220	Incidence<br>Temperature<br>Rainfall<br>Relative Humidity	(0,1,6)(0,0,0)	**0.53***	**Incidence** (LN) at MA lag 1 and 6 and first order non-seasonal differencing<br>**Min temperature**(LN) at delay 4, numerator TF order of 0 and first order non-seasonal differencing	118<br>(Jan 1998-Oct 2007)	83
Mirab Abaya	1221	Incidence<br>Temperature<br>Rainfall<br>Relative Humidity	(0,0,0)(0,0,0)	0.30	**Max temperature**(LN) at delay 4, numerator TF order of 0, denominator TF orders of 1 and 2	105<br>(Feb 1999-Oct 2007)	54
Bele	1240	Incidence<br>Rainfall	(1,0,0)(0,0,0)	0.64	**Incidence** at AR lag 1	64<br>(Jul 2002-Oct 2007)	133
Tercha *Hospital*	1335	Incidence<br>Temperature<br>Rainfall	Model ignored (Ljung-Box Q *P* value <0.05)			63<br>(Aug 2002-Oct 2007)	53
Jinka *Hospital*	1373	Incidence<br>Temperature<br>Rainfall	Model ignored (Ljung-Box Q *P* value <0.05)			62<br>(Jul 2002-Aug 2007)	126
Sawla	1380	Incidence<br>Temperature<br>Rainfall	(2,0,0)(0,0,0)	0.69	**Incidence** at AR lags 1 and 2	81<br>(Dec 2000-Aug 2007)	91

Table S2: Time series models to predict *Plasmodium falciparum* malaria incidence at different locations in south Ethiopia

Location (Health centre, unless specified)	Altitude	Available data used (Temperature- Max and Min)	Model Structure (ARIMA)	Goodness of fit (R^2, if *: stationary R^2)	Significant Variables and model description	Serial Length	Average Incidence per month
Konso Karat	1431	Incidence<br>Temperature<br>Rainfall	(1,0,0)(0,0,0)	0.94	**Incidence** at AR lag 1<br>**Rainfall** at delay 2, numerator TF orders of 0, 1 and 2, and denominator TF orders of 1 and 2.	118<br>(Jan 1998-Oct 2007)	64
Gesuba	1552	Incidence<br>Temperature<br>Rainfall	(0,0,0)(0,0,0)	-0.03	**Min temperature**(LN) at numerator TF order of 0	64<br>(Jul 2002-Oct 2007)	62
Bedesa	1609	Incidence<br>Rainfall	(1,0,0)(0,0,0)	0.65	**Incidence** at AR lag 1	53<br>(Jun 2003-Oct 2007)	65
Humbo Tebela	1618	Incidence<br>Rainfall	(0,0,0)(0,0,0)	0.19	**Rainfall** at numerator TF orders of 0, 1 and 2	64<br>(Jul 2002-Oct 2007)	98
Amaro Kele	1659	Incidence<br>Temperature<br>Rainfall	(1,0,0)(0,0,0)	0.77	**Incidence** at AR lag 1	97<br>(Sep 1999-Sep 2007)	24
Cheleklektu	1701	Incidence<br>Rainfall	(1,0,0)(0,0,0)	0.21	**Incidence** (LN) at AR lag 1	75<br>(Sep 1999-Nov 2005)	24
Wondo Genet	1742	Incidence<br>Rainfall	(0,0,2)(1,0,0)	0.97	**Incidence** at non-seasonal MA lag 2 and Seasonal AR lag 1	118<br>(Jan 1998-Oct 2007)	90
Areka	1752	Incidence<br>Rainfall	(0,1,0)(0,0,0)	**0.55***	No significant predictor	118<br>(Jan 1998-Oct 2007)	353
Alaba	1772	Incidence<br>Temperature<br>Rainfall	(0,1,1)(0,0,0)	0.46	**Incidence** (LN) at first order of non-seasonal differencing and MA lag 1<br>**Min temperature**(LN) at numerator TF order of 0, first order of non-seasonal and seasonal differencing	64<br>(Jul 2002-Oct 2007)	302
Yirg Alem	1786	Incidence<br>Temperature<br>Rainfall	Model ignored (Ljung-Box Q *P* value <0.05)			64<br>(Jul 2002-Oct 2007)	45

Table S3: Time series models to predict *Plasmodium falciparum* malaria incidence at different locations in south Ethiopia

Location (Health centre, unless specified)	Altitude	Available data used (Temperature- Max and Min)	Model Structure (ARIMA)	Goodness of fit (R^2, if *: stationary R^2)	Significant Variables and model description	Serial Length	Average Incidence per month
Kebado	1807	Incidence<br>Rainfall	Model ignored (Ljung-Box Q *P* value <0.05)			64<br>(Jul 2002-Oct 2007)	23
Wolayta Soddo	1854	Incidence<br>Temperature<br>Rainfall<br>Relative Humidity	(1,0,0)(0,0,0)	0.36	**Incidence** (LN) at AR lag 1	65<br>(Jul 2002-Nov 2007)	42
Koshe	1876	Incidence<br>Rainfall	(1,0,0)(1,1,0)	**0.91***	**Incidence** at AR lag 1 (both non-seasonal and seasonal) and first order of seasonal differencing	89<br>(Sep 1998-Jan 2006)	63
Wolkite	1884	Incidence<br>Rainfall	(0,1,0)(1,1,0)	**0.86***	**Incidence** at AR lag 1 (seasonal), non-seasonal and seasonal first order of differencing	118<br>(Jan 1998-Oct 2007)	151
Kemba	1895	Incidence<br>Rainfall	(1,0,0)(0,0,0)	0.62	**Incidence** at AR lag 1	52<br>(Jul 2003-Oct 2007)	51
Laska	1910	Incidence<br>Rainfall	(1,0,0)(0,0,0)	0.67	**Incidence** at AR lag 1	56<br>(Feb 2003-Sep 2007)	30
Aleta Wondo	1947	Incidence<br>Rainfall	(0,0,1)(0,1,0)	**0.88***	**Incidence** at MA lag 1 and first order seasonal differencing	86<br>(Sep 2000-Oct 2007)	43
Derara	1950	Incidence<br>Rainfall	(1,0,0)(0,0,0)	0.89	**Incidence** at AR lag 1	94<br>(Jan 2000-Oct 2007)	73
Shone	1959	Incidence<br>Rainfall	Model ignored (Ljung-Box Q *P* value <0.05)			64<br>(Jul 2002-Oct 2007)	98
Tora	1987	Incidence<br>Rainfall	(0,0,0)(0,0,0)	0.30	No significant predictor	60<br>(Nov 2002-Oct 2007)	69
Yirba	2023	Incidence<br>Rainfall	(1,0,0)(1,0,0)	0.68	**Incidence** at AR lag 1 (both non-seasonal and seasonal)	63<br>(Jul 2002-Sep 2007)	92

Table S4: Time series models to predict *Plasmodium falciparum* malaria incidence at different locations in south Ethiopia

Location (Health centre, unless specified)	Altitude	Available data used (Temperature- Max and Min)	Model Structure (ARIMA)	Goodness of fit (R^2, if *: stationary R^2)	Significant Variables and model description	Serial Length	Average Incidence per month
Boditi	2043	Incidence<br>Temperature<br>Rainfall	(2,0,0)(0,0,0)	0.16	**Incidence** (LN) at AR lag 1 and 2	89<br>(May 2000-Sep 2007)	98
Buee	2054	Incidence<br>Rainfall	(1,0,0)(1,0,0)	0.60	**Incidence** (LN) at AR lag 1 (both non-seasonal and seasonal)<br>**Rainfall** at delay 3 and numerator TF order of 0.	86<br>(Sep 2000-Oct 2007)	30
Butajira	2074	Incidence<br>Temperature<br>Rainfall	(1,0,0)(0,0,0)	0.94	**Incidence** at AR lag 1	118<br>(Jan 1998-Oct 2007)	50
Hossana	2306	Incidence<br>Temperature<br>Rainfall	(2,1,1)(0,0,0)	**0.66***	**Incidence** at AR lag 1 and 2, first order of differencing and MA lag 1	64<br>(Jul 2002-Oct 2007)	56
Angacha	2331	Incidence<br>Temperature<br>Rainfall	(1,0,0)(0,0,0)	0.39	**Incidence** (LN) at AR lag 1<br>**Min temperature**(LN) at delay 5 and numerator TF orders of 0	64<br>(Jul 2002-Oct 2007)	14
Arbegona	2582	Incidence<br>Temperature<br>Rainfall	(0,0,0)(0,0,0)	0.62	**Max temperature** at delay 2 and numerator TF orders of 0 and 2.<br>**Min temperature** at delay 2 and numerator TF orders of 0	51<br>(Mar 2003-May 2007)	30
Mean condition of 23 locations		Incidence<br>Rainfall	(1,0,0)(0,1,0)	**0.67***	**Incidence** at AR lag 1and first order seasonal differencing<br>**Rainfall** at delay 4 and numerator TF order of 0 and first order seasonal differencing	62<br>(Jul 2002-Aug 2007)	2579
Mean condition of 14 locations		Incidence<br>Temperature<br>±Rainfall	Model ignored (Ljung-Box Q *P* value <0.05)			51<br>(Mar 2003-May 2007)	1146
